# Object Substitution Masking in Schizophrenia: An Event-Related Potential Analysis

**DOI:** 10.3389/fpsyg.2013.00030

**Published:** 2013-02-04

**Authors:** Jonathan K. Wynn, Kristopher I. Mathis, Judith Ford, Bruno G. Breitmeyer, Michael F. Green

**Affiliations:** ^1^Veterans Affairs Greater Los Angeles Healthcare SystemLos Angeles, CA, USA; ^2^Semel Institute for Neuroscience and Human Behavior, University of CaliforniaLos Angeles, Los Angeles, USA; ^3^San Francisco Veterans Affairs Medical CenterSan Francisco, CA, USA; ^4^Department of Psychiatry, University of California San FranciscoSan Francisco, CA, USA; ^5^Department of Psychology, University of HoustonHouston, TX, USA

**Keywords:** visual processing, schizophrenia, ERP, visual backward masking, reentrant processing

## Abstract

Schizophrenia patients exhibit deficits on visual processing tasks, including visual backward masking, and these impairments are related to deficits in higher-level processes. In the current study we used electroencephalography techniques to examine successive stages and pathways of visual processing in a specialized masking paradigm, four-dot masking, which involves masking by object substitution. Seventy-six schizophrenia patients and 66 healthy controls had event-related potentials (ERPs) recorded during four-dot masking. Target visibility was manipulated by changing stimulus onset asynchrony (SOA) between the target and mask, such that performance decreased with increasing SOA. Three SOAs were used: 0, 50, and 100 ms. The P100 and N100 perceptual ERPs were examined. Additionally, the visual awareness negativity (VAN) to correct vs. incorrect responses, an index of reentrant processing, was examined for SOAs 50 and 100 ms. Results showed that patients performed worse than controls on the behavioral task across all SOAs. The ERP results revealed that patients had significantly smaller P100 and N100 amplitudes, though there was no effect of SOA on either component in either group. In healthy controls, but not patients, N100 amplitude correlated significantly with behavioral performance at SOAs where masking occurred, such that higher accuracy correlated with a larger N100. Healthy controls, but not patients, exhibited a larger VAN to correct vs. incorrect responses. The results indicate that the N100 appears to be related to attentional effort in the task in controls, but not patients. Considering that the VAN is thought to reflect reentrant processing, one interpretation of the findings is that patients’ lack of VAN response and poorer performance may be related to dysfunctional reentrant processing.

## Introduction

Patients with schizophrenia exhibit several visual processing impairments (Green et al., 1994; Butler et al., 2003; Butler and Javitt, 2005; Rassovsky et al., 2005; Wynn et al., 2005; Silverstein and Keane, 2011), and these impairments have been tied to specific neural abnormalities, such as magnocellular pathway dysfunction, NMDA functioning, and activation in the lateral occipital complex (Butler et al., 2005; Green et al., 2009; Javitt, 2009). However, it is unclear whether visual processing impairment is isolated to early or later processing stages (Javitt, 2009; Dias et al., 2011; Rassovsky et al., 2011). Visual processing divides roughly into two stages: (1) initial formation of a percept, and (2) subsequent processing of the percept until it reaches awareness (Di Lollo et al., 2000; Enns and Di Lollo, 2000; Ro et al., 2003; Enns, 2004; Chen and Treisman, 2009; Dux et al., 2010). The first stage is thought to involve a neural feedforward sweep from retina to primary visual cortex. The second stage involves reentrant processes (i.e., recurrent cortico-cortical circuits) that refine the initially ambiguous percept until it becomes recognizable. These reentrant processes have even been shown to feedback to very early neural areas such as the lateral geniculate nucleus (Sillito et al., 1994).

Visual backward masking can be used to assess both of these stages. Masking that disrupts feedforward processing usually occurs between 0 and 100 ms after target onset. Masking at the reentrant stage occurs slightly later (starting approximately 100 ms after stimulus onset; Woodman and Luck, 2003; Prime et al., 2011), and results in the mask displacing the neural representation of the target, a phenomenon termed object substitution masking (Di Lollo et al., 2000; Enns and Di Lollo, 2000; Chen and Treisman, 2009; Dux et al., 2010). The premise underlying object substitution is that the feedforward sweep of the target must be refined at higher cortical levels through reentrant processes to the lower input levels. If the mask is presented before the target is identified at this higher-level, there will be a mismatch between the feedforward and reentrant sweeps, and processing will switch to the mask. In essence, the target may be correctly identified at some level within the primary visual processing stream but accurate information of that target is not passed on to higher-level visual processing systems. Object substitution masking, therefore, has provided a method to examine reentrant visual processing (Woodman and Luck, 2003).

Four-dot masking is a paradigm that is thought to act through object substitution (Enns and Di Lollo, 1997; Di Lollo et al., 2000; Enns, 2004). In four-dot masking, performance is greatest when the target and the mask have the same onset and offset (effectively cuing the observer to the target). Four-dot masking can occur when both target and mask have the same onset (termed “common-onset masking”) and the mask remains visible longer than the target and can also occur when the onset of the mask appears at varying stimulus onset asynchronies (SOAs) relative to the target (Enns and Di Lollo, 2000). Using this second type of four-dot masking in a behavioral study we found impairments in schizophrenia patients (Green et al., 2011). Moreover, it did not appear that the patients’ deficit was due to issues in initial processing of the target because when using the same SOAs and using a single dot to indicate which target to identify, we found: (1) that there was no group difference in behavior; and (2) iconic decay of the target was not different between groups. Therefore, the increased masking effects in schizophrenia patients appeared to be due to the presence of the mask and not other factors. However, we could not determine with a behavioral task if deficits in schizophrenia patients in the object formation stage of visual processing contributed to the deficits assessed with four-dot masking. Indeed, the version of four-dot masking that we used (with delayed-onset mask) could have involved some early masking of object formation, in addition to its characteristic object substitution masking. With the excellent temporal resolution of electroencephalography (EEG) and event-related potentials (ERPs) it is possible to evaluate perceptual and post-perceptual stages separately.

Specific ERP waveform components track with stages of visual information processing, including the visual P100 and N100 and the visual awareness negativity (VAN). The P100 is a positive wave peaking approximately 100 ms post stimulus onset. Several neural generators of the P100 have been identified, with activity largest over dorsal stream sites, with extrastriate and striate contributions (Maier et al., 1987; Aine et al., 1995; Di Russo et al., 2001; Vanni et al., 2004). The N100 is a negative wave peaking approximately 150 ms post stimulus onset. Multiple generators have also been identified for the N100, with the largest activity seen in ventral stream structures such as the object-sensitive lateral occipital complex (LOC; e.g., Bentin et al., 1999; Doniger et al., 2000, 2001, 2002). The VAN has been reported to appear as a relative increase in negativity to conscious vs. unconscious (e.g., correct vs. incorrect) stimuli appearing approximately 200–300 ms in occipito-temporal sites, though there is considerable variability as to when this component peaks (for a review, see (Railo et al., 2011). Moreover, this ERP component has been proposed as a measure of reentrant cortical activity (Koivisto et al., 2006; Wilenius and Revonsuo, 2007) because of its regional distribution, it is associated with awareness of a visual target and its latency falls within the time frame of when reentrant cortical activity is thought to occur (Lamme and Roelfsema, 2000; Di Russo et al., 2001).

Several ERP studies have examined backward masking in healthy participants. Fahrenfort et al. (2007) found that both masked and unmasked targets produced a strong bilateral anterior occipito-temporal activation that occurred prior to 110 ms (likely consistent with the visual P100), which the authors attributed to feedforward processing of visual stimuli. However, only unmasked trials resulted in activity between 110–140 ms at posterior occipital sites, with no activity for masked trials seen at this time frame. The authors interpreted these findings as reflecting reentrant processing being interrupted by the presence of the mask. With a backward masking procedure similar to Fahrenfort et al; Van Loon et al. (2012) found that masking had no effect on the earliest ERP components (i.e., <120 ms, corresponding to the P100) but effectively decreased later ERP components (i.e., >150 ms, corresponding to the N100). Moreover, only the later, N100-like response correlated with behavior, such that greater accuracy correlated with a larger N100.

To date, only a handful of ERP studies of four-dot masking have been published (Woodman and Luck, 2003; Reiss and Hoffman, 2006; Kotsoni et al., 2007; Prime et al., 2011) and these studies evaluated whether four-dot masking is consistent with object substitution. In summary, these studies found that feedforward processing of an object is left intact, but later reentrant processing of the target is interrupted due to the mask substituting the target during this stage.

In the current study, we tracked the time course of visual information processing during object substitution masking in a large sample of schizophrenia patients and healthy controls. We manipulated visibility of the targets using various SOAs, such that performance is best at an SOA of 0 ms, with performance decreasing at higher SOAs. We chose this method of delayed-onset masking, rather than common-onset masking, to more directly compare the results of the current study to other masking paradigms commonly used in schizophrenia research that have delayed-onset masks (e.g., see Green et al., 2011). We used ERPs to assess early and later perceptual stages (P100 and N100) and the VAN to examine activity thought to be directly related to reentrant processing. We used the exact same paradigm as in our behavioral study that showed patient–control differences, though sampled a fewer number of SOAs. Based on prior EEG studies of backward masking, we hypothesized that the VAN would be the first component sensitive to object substitution masking. In particular, we expected that in healthy controls a larger VAN to correct vs. incorrect responses would be seen, whereas patients would not exhibit this effect.

## Materials and Methods

### Participants

Most participants also participated in a behavioral study of object substitution in a separate session and using a somewhat different procedure from the one presented in the current paper (Green et al., 2011). Seventy-seven stabilized outpatients with schizophrenia and 66 healthy control subjects participated in the study. One patient was excluded from analysis due to having an insufficient amount of usable EEG data (see below). Thus, the final patient sample size was *n* = 76. Patients were recruited from outpatient treatment clinics at the Veterans Affairs Greater Los Angeles Healthcare System (VAGLAHS) and through presentations at community residences. Patients met criteria based on the Structured Clinical Interview for DSM-IV Axis I Disorders (First et al., 1997). Sixty-two patients were receiving atypical antipsychotic medications, seven were receiving typical antipsychotic medications, three were receiving both types of medication, and four were not taking an antipsychotic medication at time of assessment.

Healthy control participants were recruited through internet and newspaper advertisements. Control participants were screened with the SCID and SCID–II (First et al., 1996) and were excluded if they met criteria for any lifetime psychotic disorder; bipolar mood disorder; recurrent depression; substance dependence; paranoid, schizotypal, or schizoid personality disorder; or any evidence (according to participant report) of a history of psychotic disorder among their first-degree relatives.

Additional exclusion criteria for both groups included being younger than 18 or older than 60 years, diagnosed with an active substance use disorder in the past 6 months, any identifiable neurological disorder, mental retardation, history of loss of consciousness for more than 1 h, or insufficient fluency in English. All participants had the capacity to give informed consent and provided written informed consent after all procedures were explained in accordance with procedures approved by the Institutional Review Boards at UCLA and VAGLAHS.

### Clinical ratings

Psychiatric symptoms during the previous month were rated using the 24-item UCLA version of the Brief Psychiatric Rating Scale (BPRS; Overall and Gorham, 1962; Lukoff et al., 1986) and the Scale for the Assessment of Negative Symptoms (SANS; Andreasen, 1984) by a trained rater. For the BPRS we report the “positive symptom” and “depression/anxiety” factors (Kopelowicz et al., 2008); for the SANS we report the global scores for affective flattening, alogia, anhedonia, and avolition (Table 1). All clinical assessments were conducted by interviewers trained to reliability through the Treatment Unit of the Department of Veterans Affairs VISN 22 Mental Illness Research, Education, and Clinical Center (MIRECC) based on previously reported procedures (Ventura et al., 1993, 1998).

**Table 1:** **Demographic information and symptom ratings**.

	*Patients (n* = *76)*		*Normal controls (n* = *66)*	
	Mean	SD	Mean	SD
Age*	46.3	10.1	37.7	9.8
Parental education	12.8	2.9	13.5	2.5
Percent female	20%		27%	
**BPRS**
Total score	43.6	9.9		
Factors (mean score per item)
Depression/anxiety	1.9	0.7	
Positive symptoms	2.2	0.9	
**SANS global scores**
Affective flattening	1.6	1.3		
Alogia	0.7	1.0	
Avolition	2.2	1.4	
Anhedonia	2.7	1.8	

*p < 0.01, for difference between controls and patients.

### Backward masking task

All stimuli were presented using E-Prime 1.1 (Psychological Software Tools, Pittsburgh, PA) on a 17″ cathode ray tube monitor running at a 160 Hz refresh rate. Participants sat 1 m away from the monitor.

**Figure 1:**
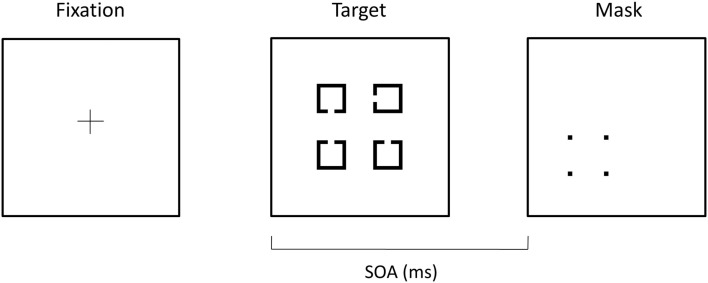
**Example of stimuli used in the four-dot backward masking task**. A fixation cross was shown for 450 ms followed by a 500 ms blank screen. Potential targets, consisting of squares with notches cut in the top, left side, or bottom, were then shown for 25 ms. A mask, consisting of four-dots that surrounded one of the potential targets, was presented for 37.5 ms. The mask served as the cue to the participant as to which target they were to identify the direction of the notch.

The four-dot masking procedure was modified from similar tasks described elsewhere (Enns and Di Lollo, 2000; Enns, 2004) and was based on a larger behavioral study of four-dot masking from our laboratory (Green et al., 2011). In the task, four potential targets, consisting of four squares with a notch missing from the top, bottom, or side, appeared in a notional square on the monitor. Following the target stimuli, a mask was presented which comprised four-dots arranged in a square that surrounded, but did not touch, one of the potential targets (see Figure 1). The mask cued the location of the target. Each potential target measured 1.55° × 1.55° of visual angle and was arranged in a square of 4.58° × 4.58° of visual angle. The four-dot mask measured 2.23° × 2.23° of visual angle and each dot in the mask subtended 0.23° × 0.23° of visual angle. The target array was presented for 25 ms and the mask was presented for 37.5 ms. All stimuli were suprathreshold, with stimuli presented in black presented on a white background. The luminance of the background was 260 lx while that of the stimuli was 9 lx, resulting in a contrast of ∼93%, defined by Michelson’s contrast: contrast = (*L*_max_−*L*_min_)/(*L*_max_ + *L*_min_). We collected data on four target-mask stimulus onset asynchronies (SOAs: 0, 50, 100, and 150 ms). However, we only analyzed data from the first three SOAs, as the mask terminated on or before the ERP components of interest for these SOAs, and there was little difference in performance between SOAs of 100 and 150. Fifty-four trials per SOA were presented in quasi-randomized fashion (18 trials for each of the three potential sides where the notch appears).

Each trial started with a fixation cross presented for 450 ms followed by a blank screen for 500 ms. Target and mask stimuli, separated by the SOAs mentioned above, were then presented. A 1 s blank screen was then presented, followed by a prompt for the participant to make a response. Participants verbally reported the direction of the notch in the target and the experimenter entered the response and initiated the next trial. The total number of correct responses (out of 54) at each SOA was analyzed.

### EEG recording

Electroencephalography was continuously recorded using a 64-channel Neuroscan SynAmps2 amplifier and a Neuroscan 64-channel QuickCap (Compumedics USA, Charlotte, NC). Data were sampled at 500 Hz with a bandpass of 1–100 Hz. Horizontal electrooculogram (EOG; placed on the outer canthus of the left and right eye) and vertical EOG (placed above and below the left eye) was also recorded. The reference during recording was a point halfway between electrodes Cz and CPz, and all sites were re-referenced offline to the average of the left and right mastoids. An electrode affixed to the forehead served to ground the array.

### ERP data analysis

Data were processed offline using Neuroscan Scan 4.3 and BrainVision Analyzer 2 software (Brain Products, Gilching, Germany). Vertical eye blinks were removed using a regression-based algorithm (Semlitsch et al., 1986). Data were low-pass filtered at 30 Hz, with a 24 dB roll off zero phase shift filter. Data were epoched to 100 ms pre- and 700 ms post-target onset. Baseline correction from the 100 ms prior to stimulus presentation was applied. Epochs containing activity that exceeded ±100 μV were rejected at the sites that were used for data analysis (P7, P5, P6, P8, PO7, PO8, O1, O2). Visual inspection of trials was then performed to eliminate any remaining abnormal EEG responses. The mean number of valid trials per SOA (out of a total of 54) included in subsequent statistical analyses was 51.7 (range for individual participants, 29–54) and 52.4 (range for individual participants, 44–54) for patients and controls, respectively.

Event-related potentials were created by averaging together all accepted trials (regardless of accuracy), separately for each SOA. A time window was defined for each ERP component based on the peak activity observed by inspection of the mean global field power averaged across all subjects and SOAs. The width of the time window was selected to ensure coverage of each component and the mean activity within each window was the main dependent measure. The time windows were 68–108 and 134–174 ms for the P100 and N100, respectively. The P100 and N100 were examined in six parieto-occipital electrode sites where visual ERPs were largest based on visual inspection of the waveforms (P7, PO7, P5, P8, PO8, and P6). These sites were also chosen as they overlap with sites used in previous examinations of the N100 and P100 response in visual processing studies in schizophrenia (e.g., Butler et al., 2007). Activity was examined separately for each hemisphere by averaging the three left and the three right electrodes.

The VAN was analyzed in 50 patients and 43 healthy controls that had at least 15 artifact-free epochs for both correct and incorrect responses at each SOA of 50 and 100 ms. For patients, there were a mean (SD) 24.1 (5.6) and 28.0 (5.5) trials for correct and incorrect, respectively, averaged over the two SOAs; for controls there were 28.6 (6.2) and 24.2 (6.1) trials for correct and incorrect, respectively. Activity at electrodes PO7, PO8, O1, and O2 were examined for the VAN. The VAN was measured as the mean activity within the time window of 250–310 ms. Additionally, the P100 and N100 (using the same time windows and electrodes described above) to correct and incorrect responses were examined.

### Data analysis

For demographic data, group differences were evaluated with *t*-tests and chi-square tests. 2 (group) × 2 (hemisphere) × 3 (SOA) repeated measures ANOVAs were run separately for P100 and N100 for the entire sample, disregarding accuracy. To examine the VAN and accuracy effects on the P100 and N100 in the subsample, separate 2 (group) × 2 (accuracy) × 2 (SOA) repeated measures ANOVAs were run. In cases of repeated measures with more than one degree of freedom, we used Greenhouse–Geisser correction factors (ε). We report the uncorrected degrees of freedom, the corrected *p*-value, partial eta-square effect sizes ($\eta_{p}^{2}$), and ε. All statistical analyses used a two-tailed significance level of 0.05. Bonferroni-corrected multiple comparisons were used to ensure a family wise significance level of *p* < 0.05.

## Results

### Demographics

Demographic information is presented in Table 1. Patients were clinically stable with relatively low levels of symptoms. They were significantly older than controls but did not differ in terms of gender or parental education. As we have shown in our previous studies, age is significantly correlated with backward masking performance (Green et al., 2011). Age is also largely correlated with performance in the current study for both groups, with *r*’s between −0.30 and −0.38 for each SOA. Therefore, we included age as a covariate in our analyses. We report age-corrected means (standard deviations) where appropriate.

### Behavior

**Figure 2:**
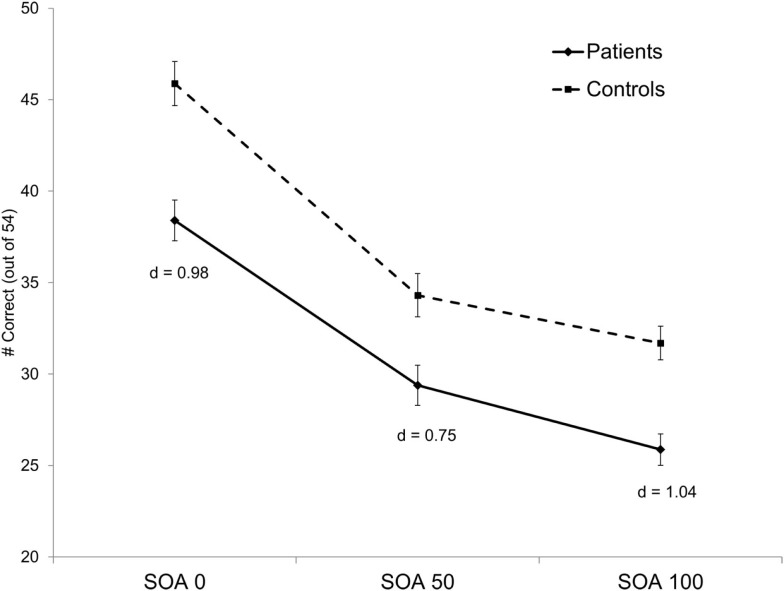
**Behavioral performance for each group on the four-dot backward masking task**. Groups differed significantly across all SOAs. Bars represent ± one standard error. Cohen’s d effect sizes for between-group differences are presented at each SOA.

Results of the analysis revealed a significant main effect of group (F_1, 139_ = 19.76, *p* < 0.001, $\eta_{p}^{2}$ = 0.12) and a significant main effect of SOA (F_2, 278_ = 8.48, *p* <  0.001, ε = 0.92, $\eta_{p}^{2}$ = 0.06). The group by SOA interaction was not significant (*p* > 0.17). Overall, patients showed poorer performance compared to controls and both groups showed the expected decline in accuracy as SOA increased (see Figure 2).

### EEG

**Figure 3:**
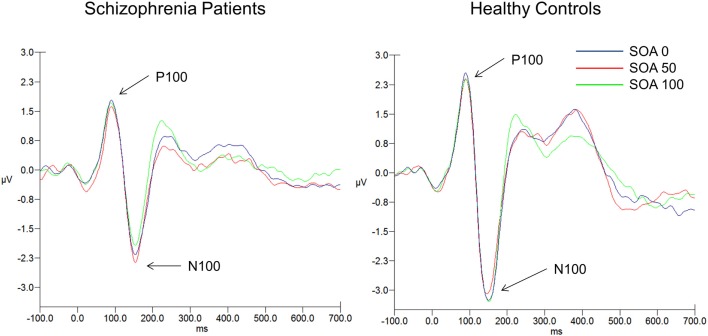
**ERPs time-locked to target onset (0 ms) for both groups at each SOA**. The P100 and N100 (averaged over electrodes P7, PO7, P5, P6, PO8, P8) are noted by arrows. SOA 0 ms, dark blue; SOA 50 ms, red; SOA 100 ms, green.

**Figure 4:**
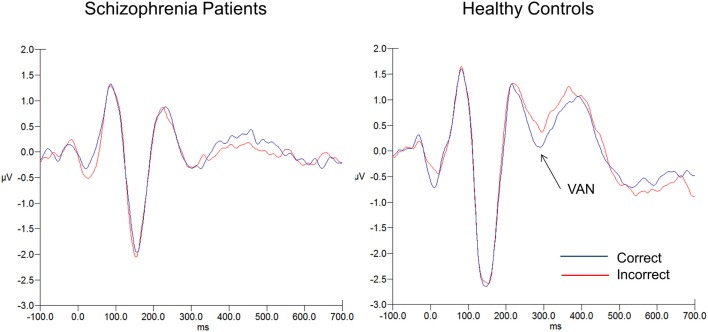
**VAN waveforms for correct (blue) and incorrect (red) responses for schizophrenia patients (on the left) and healthy controls (on the right)**. The VAN was averaged over SOAs 50 and 100 (averaged over electrodes PO7, PO8, O1, O2).

Means and SD for the two ERP components can be seen in Table 2. Figure 3 shows the ERP waveforms (averaged over the six parieto-occipital electrodes mentioned above) at each SOA for each group. Figure 4 shows the VAN waveforms (averaged over the two SOAs and the four parieto-occipital electrodes mentioned above) for each group.

**Table 2:** **Mean (SD) amplitudes for each group and each ERP component**.

		SOA 0	SOA 50	SOA 100
P100	Patients			
	L	1.37 (1.56)	1.24 (1.57)	1.38 (1.52)
	R	1.68 (1.68)	1.66 (1.74)	1.75 (1.79)
	Controls			
	L	1.89 (1.57)	1.79 (1.59)	1.70 (1.54)
	R	2.03 (1.69)	1.98 (1.76)	1.89 (1.81)
N100	Patients			
	L	−1.76 (2.43)	−1.93 (2.33)	−1.60 (2.23)
	R	−2.19 (2.54)	−2.09 (2.53)	−1.85 (2.40)
	Controls			
	L	−2.55 (2.46)	−2.38 (2.36)	−2.67 (2.25)
	R	−3.09 (2.57)	−2.74 (2.56)	−3.08 (2.43)

*L, left; R, right*.

#### P100

There was only a significant effect for group (F_1, 139_ = 3.94, *p* < 0.05, $\eta_{p}^{2}$ = 0.03). There were no other significant main effects or interactions. Controls had a larger P100 compared to patients [1.92 (1.26) versus 1.41 (1.25)μV, respectively].

#### N100

Results of the analysis revealed a significant main effect of group (F_1,139_ = 5.42, *p* < 0.03, $\eta_{p}^{2}$ = 0.04) and a significant group × SOA interaction (F_2, 278_ = 7.81, *p* < 0.001, ε = 0.98, $\eta_{p}^{2}$ = 0.05). Controls had a significantly larger N100 compared to patients [−2.86(2.13) versus −1.77(2.01)μV]. Controls showed significantly larger N100 amplitudes at all SOAs, except at SOA 50, compared to the patients. The group × SOA interaction was due to patients showing an increase in amplitude at SOA 50 whereas controls show a decrease, a pattern difference that was not predicted.

### Effects of accuracy on ERPs at SOAs 50 and 100 ms

#### P100

The main effects of group, accuracy, and SOA were not significant; there were also no significant interactions, all *F*’s < 1.40, *p*’s > 0.24.

#### N100

The main effects of group, accuracy, and SOA were not significant. There was only a significant group × SOA interaction (F_1, 90_ = 11.25, *p* <  0.001, $\eta_{p}^{2}$ = 0.11). The group × SOA interaction was due to patients and controls having similar amplitudes at an SOA of 50, whereas controls had a larger amplitude than patients at an SOA of 100 ms.

#### VAN

As the VAN appears as a greater difference between correct vs. incorrect responses, we expected to see a significant difference in the healthy controls, and we did. For controls, the difference between correct and incorrect reached significance (t_42_ = 2.47, *p* < 0.02), 0.27 (1.25) vs. 0.59 (1.15) μV, correct vs. incorrect respectively (effect size = 0.27). In contrast, for the patients the *t*-tests showed no significant difference for correct vs. incorrect responses in patients (t_49_ = 0.55, *p* < 0.60), −0.15 (0.94) vs. −0.08 (1.03) μV, correct vs. incorrect respectively (effect size = 0.07). The group × accuracy interaction failed to reach significance (F_1, 90_ = 2.18, *p* < 0.15, $\eta_{p}^{2}$ = 0.02). Hence, although group differences in VAN were predicted, they need to be interpreted with caution given the non-significant group by accuracy interaction.

### Relationship between ERPs and Behavior

Correlations were examined between the P100 and N100 and behavioral performance at each SOA for each group, controlling for age. P100 and N100 activity was averaged across hemisphere for each SOA. As can be seen in Table 3, P100 amplitude was largely uncorrelated with performance in both groups (only one of the eight correlations reached significance). N100 amplitude correlated significantly with performance at the two later SOAs (where masking effects occurred), but only in the healthy controls. P100 was significantly correlated with N100 at each respective SOA in both groups, *r*’s ranging from −0.50 to −0.33.

**Table 3:** **Correlations between backward masking performance (# targets correctly identified) and ERP components**.

	SOA0	SOA50	SOA100
**Schizophrenia patients**			
P100	0.10	0.10	0.13
N100	−0.19	−0.13	−0.03
**Healthy controls**			
P100	0.09	0.09	0.29*
N100	−0.14	−0.38*	−0.31*

*p < 0.05.

### Age effects

Because analyses of covariance can sometimes misrepresent relationships among variables, we considered the potential effect of age on the behavioral and ERP data, without including a covariance analysis. We performed a median split, creating a “young” patient group (*n* = 39, mean age = 38.6) and an “old” patient group (*n* = 37, mean age = 54.4). The “young” patient group more closely matched the age of the healthy control group, with no statistical difference in age between these two groups. Two analyses were conducted. First, differences in ERPs and behavior between the “young” and “old” schizophrenia patient groups were examined; no significant group effects or interactions with group were seen (all *F*’s < 1.0, *p*’s > 0.4). Second, differences in ERPs and behavior between only the “young” patients and the healthy controls were examined. Similarly, we performed this same split for the VAN analyses, with a “young” patient group (*n* = 25, mean age = 40.0) and an “old” patient group (*n* = 25, mean age = 50.5) being created. Again, there were no difference between the “young” and “old” patient groups and the pattern of results remained the same comparing the “young” patients to the healthy control sample. These analyses revealed the exact same pattern of results as when using the entire patient sample, indicating the presence of behavioral and ERP differences between schizophrenia patients and healthy controls regardless of age.

## Discussion

Using ERPs, we were able to clarify the nature of the patients’ behavioral deficit in four-dot object substitution masking. Several key findings emerged from this study. First, patients exhibited significantly reduced P100 and N100 amplitudes compared to controls across all SOAs, indicating impairment in perceptual processes across levels of visibility. Second, N100 correlated with accuracy in controls, but not patients, at SOAs where masking occurs. Third, controls, but not patients, exhibited a VAN response, though the group by accuracy interaction did not reach significance. These results provide further evidence that schizophrenia patients likely have a dysfunction in reentrant visual processing.

The reduced amplitude of P100 and N100 is consistent with previous reports of EEG abnormalities during early visual perception in schizophrenia (Foxe et al., 2001; Doniger et al., 2002; Schechter et al., 2005), suggesting dysfunction at the earliest stages of object formation. However, it seems unlikely that the P100 component directly affected object substitution for either group (i.e., amplitudes did not change with changing visibility). As mentioned in the introduction, ERP studies of backward masking show no effect on the P100, indicating that feedforward processing is left relatively intact. Furthermore, in the accuracy analysis of the ERP data, there was no effect of accuracy on the P100 and the subgroup of patients and controls included in the accuracy analyses did not differ in P100 amplitude. We found a somewhat mixed pattern for the effects of object substitution masking on the N100, in that there were no effects of SOA (i.e., visibility) and no effect of accuracy on N100 amplitude. On the other hand, there was a correlation between behavior and amplitude in the controls that may simply reflect greater attentional effort devoted to processing stimuli that were harder to accurately detect.

The finding of the lack of a VAN to correct vs. incorrect trials in schizophrenia patients points to dysfunctional reentrant processing. As mentioned in the introduction, the VAN appears to be the first ERP correlate of visual consciousness and likely is a direct measure of reentrant processing. Moreover, there was no effect of accuracy on either the P100 or N100, reflecting that these components are unlikely related to reentrant processing. The healthy controls exhibited a VAN, indicating that effective four-dot masking in this study interrupted reentrant visual processing. Given the lack of this finding in the schizophrenia patients, these results imply that schizophrenia patients’ deficit during object substitution may be due to a type of dysfunction (e.g., lack of neural synchronization, coherence, etc.) in reentrant processing along this pathway. Regardless of how patients correctly identified stimuli in the current study, the VAN results suggest a dysfunction in reentrant processing. However, the lack of a significant group × accuracy interaction in the VAN data means that we interpret these findings with caution.

There also remains the possibility that other processes, such as consciousness of stimuli and attentional effort, may be accounting for our behavioral and EEG findings. For example, Del Cul et al. (2006) found that schizophrenia patients’ ability to consciously process targets in the presence of a mask was diminished in comparison to healthy controls; however, their ability to subliminally process the targets was intact. The authors interpreted their findings as showing that patients’ feedforward processing during masking was intact whereas conscious processing of those stimuli are dysfunctional. Also, Lalanne et al. (2012) suggested that backward masking impairments in schizophrenia patients may be due to a lack of focused attention on the target. Finally, using pupillometry during a backward masking task, Granholm et al. (2009) found that impaired performance in patients was attributable to abnormalities in attentional resource availability. These other factors may be alternative possibilities for the findings, or constitute explanations at other levels of processing.

As with our behavioral study (Green et al., 2011), patients’ behavioral performance was worse than controls across all SOAs, including at the earliest SOA that has minimal masking. This finding limits interpretation in that it could be that patients are unable to correctly identify the target regardless of the effectiveness of the mask. We were able to address this possibility in our previous behavioral study which included a cuing procedure (see Introduction). For example, four-dot masking tasks depend on iconic decay (the visible persistence of a target) and group differences in this process could account for the masking deficit. However, we examined iconic decay in our behavioral study using a simple cuing task and found no differences between the patients and controls on iconic decay. Thus, differences in decay rates are unlikely to account for the performance differences. The current paradigm had no similar control procedure, and that is one limitation of the study.

The current study has other limitations. First, nearly all patients were medicated at the time of testing, potentially affecting the results. However, there is evidence that antipsychotic medication does not affect masking performance (Cadenhead et al., 1997; Green et al., 1999; Butler et al., 2003) or visual ERPs (Butler et al., 2001; Schechter et al., 2005), making medication effects unlikely. Second, our delayed-onset paradigm might not have completely eliminated the interrupting effect of the onset of the transient channels elicited by the mask (Jannati et al., 2011). While a common-onset masking procedure (Enns and Di Lollo, 2000) might be a more complete way to isolate reentrant processing during masking, there remains considerable evidence that even in metacontrast masking reentrant processing is being interrupted (Di Lollo et al., 2000; Breitmeyer, 2007; Fahrenfort et al., 2007). Therefore it is likely that our behavioral and VAN results reflect in a dysfunction in reentrant processing in patients with schizophrenia.

Another limitation is that our design was not optimized for eliciting P300 or N2pc that have been examined in the four-dot backward masking studies conducted in healthy controls (Woodman and Luck, 2003; Prime et al., 2011). Specifically, we did not have a rare stimulus, we had SOAs that were closely spaced, and we did not have lateralized presentation of the stimuli that would have enabled us to collect these other components. A small P300-like component is visible for the shortest SOA in Figure 3, but due to our parameters we did not consider it to be a valid P300. Finally, we did not equate the groups for their “unmasked” target performance (i.e., performance at an SOA of 0 ms), as we have done in our previous backward masking studies (Green et al., 2002, 2003, 2006).

The results from this study help clarify findings from our previous behavioral study on object substitution masking (Green et al., 2011). It was not possible to determine from the behavioral study (particularly given our choice of a delayed-onset mask) whether the deficits in the patients’ identification of targets were due to impairment at the earliest stage of object formation or to impairment in a later stage where reentrant processes are necessary to refine the visual percept. The current study offers tentative support for the idea that impairment of visual processing was attributable to deficits associated with reentrant processing after the initial feedforward sweep. While there is abundant evidence, from our laboratory and others, that dysfunctional bottom-up processes in schizophrenia affect downstream processing of higher-level tasks (Sergi and Green, 2003; Leitman et al., 2005; Dias et al., 2011; Pinheiro et al., 2012), our findings with this paradigm implicate a slightly later visual processing stage.

## Conflict of Interest Statement

The authors declare that the research was conducted in the absence of any commercial or financial relationships that could be construed as a potential conflict of interest.

## References

[B1] Aine C. J. Supek S. George J. S. (1995). Temporal dynamics of visual evoked neuromagnetic sources: effects of stimulus parameters and selective attention. Int. J. Neurosci. 80, 79–104 10.3109/00207459508986095 7775063

[B2] Andreasen N. C. (1984). The scale for the assessment of negative symptoms (SANS). Iowa City, IA: The University of Iowa

[B3] Bentin S. Mouchetant-Rostaing Y. Giard M. H. Echallier J. F. Pernier J. (1999). ERP manifestations of processing printed words at different psycholinquistic levels: time course and scalp distribution. J. Cogn. Neurosci. 11, 235–260 10.1162/089892999563373 10402254

[B4] Breitmeyer B. G. (2007). Visual masking: past accomplishments, present status, future developments. Adv. Cogn. Psychol. 3, 9–20 10.2478/v10053-008-0010-7 20517494 PMC2864971

[B5] Butler P. D. Desanti L. A. Maddox J. Harkavy-Friedman J. M. Amador X. F. Goetz R. R. (2003). Visual backward-masking deficits in schizophrenia: Relationship to visual pathway function and symptomatology. Schizophr. Res. 59, 199–209 10.1016/S0920-9964(01)00341-3 12414076

[B6] Butler P. D. Javitt D. C. (2005). Early-stage visual processing deficits in schizophrenia. Curr. Opin. Psychiatry 18, 151–157 10.1097/00001504-200503000-00008 16639168 PMC1994776

[B7] Butler P. D. Martinez A. Foxe J. J. Kim D. Zemon V. Silipo G. (2007). Subcortical visual dysfunction in schizohprenia drives secondary cortical impairments. Brain 130, 417–430 10.1093/brain/awm154 16984902 PMC2072909

[B8] Butler P. D. Schechter I. Zemon V. Schwartz S. G. Greenstein V. C. Gordon J. (2001). Dysfunction of early-stage visual processing in schizophrenia. Am. J. Psychiatry 158, 1126–1133 10.1176/appi.ajp.158.7.1126 11431235

[B9] Butler P. D. Zemon V. Schechter I. Saperstein A. M. Hoptman M. J. Lim K. O. (2005). Early-stage visual processing and cortical amplification deficits in schizophrenia. Arch. Gen. Psychiatry 62, 495–504 10.1001/archpsyc.62.5.495 15867102 PMC1298183

[B10] Cadenhead K. S. Geyer M. A. Butler R. W. Perry W. Sprock J. Braff D. L. (1997). Information processing deficits of schizophrenia patients: Relationship to clinical ratings, gender and medication status. Schizophr. Res. 28, 51–62 10.1016/S0920-9964(97)00085-6 9428064

[B11] Chen Z. Treisman A. (2009). Implicit perception and level of processing in object-substitution masking. Psychol. Sci. 20, 560–567 10.1111/j.1467-9280.2009.02328.x 19368699

[B12] Del Cul A. Dehaene S. Leboyer M. (2006). Preserved subliminal processing and impaired conscious access in schizophrenia. Arch. Gen. Psychiatry 63, 1313–1323 10.1001/archpsyc.63.12.1313 17146006 PMC2901353

[B13] Di Lollo V. Enns J. T. Rensink R. A. (2000). Competition for consciousness among visual events: The psychophysics of reentrant visual processes. J. Exp. Psychol. Gen. 129, 481–507 10.1037/0096-3445.129.4.481 11142864

[B14] Di Russo F. Martinez A. Sereno M. I. Pitzalis S. Hillyard S. A. (2001). Cortical sources of the early components of the visual evoked potential. Hum. Brain Mapp. 15, 95–111 10.1002/hbm.10010 11835601 PMC6871868

[B15] Dias E. C. Butler P. D. Hoptman M. J. Javitt D. C. (2011). Early sensory contributions to contextual encoding deficits in schizophrenia. Arch. Gen. Psychiatry 68, 654–664 10.1001/archgenpsychiatry.2011.17 21383251 PMC4346148

[B16] Doniger G. M. Foxe J. J. Murray M. M. Higgins B. A. Javitt D. C. (2002). Impaired visual object recognition and dorsal/ventral stream interaction in schizophrenia. Arch. Gen. Psychiatry 59, 1011–1020 10.1001/archpsyc.59.11.1011 12418934

[B17] Doniger G. M. Foxe J. J. Murray G. M. Higgins B. A. Snodgrass J. G. Schroeder C. E. (2000). Activation timecourse of ventral visual stream object-recognition areas: High-density electrical mapping of perceptual closure processes. J. Cogn. Neurosci. 12, 615–621 10.1162/089892900562372 10936914

[B18] Doniger G. M. Foxe J. J. Schroeder C. E. Murray G. M. Higgins B. A. Javitt D. C. (2001). Visual perceptual learning in human object recognition areas: a repetition priming study using high-density electrical mapping. Neuroimage 13, 305–313 10.1006/nimg.2000.0684 11162271

[B19] Dux P. E. Visser T. A. Goodhew S. C. Lipp O. V. (2010). Delayed reentrant processing impairs visual awareness: An object-substitution masking study. Psychol. Sci. 21, 1242–1247 10.1177/0956797610379866 20696853

[B20] Enns J. T. (2004). Object substitution and its relation to other forms of visual masking. Vision Res. 44, 1321–1331 10.1016/j.visres.2003.10.024 15066393

[B21] Enns J. T. Di Lollo V. (1997). Object substitution: A new form of masking in unattended visual locations. Psychol. Sci. 8, 135–139 10.1111/j.1467-9280.1997.tb00696.x

[B22] Enns J. T. Di Lollo V. (2000). What’s new in visual masking? Trends Cogn. Sci. 4, 345–352 10.1016/S1364-6613(00)01520-5 10962616

[B23] Fahrenfort J. J. Scholte H. S. Lamme V. A. (2007). Masking disrupts reentrant processing in human visual cortex. J. Cogn. Neurosci. 19, 1488–1497 10.1162/jocn.2007.19.9.1488 17714010

[B24] First M. B. Gibbon M. Spitzer R. L. Williams J. B. W. Benjamin L. (1996). Structured Clinical Interview for DSM-IV Avis II Personality Disorders. New York: New York State Psychiatric Institute

[B25] First M. B. Spitzer R. L. Gibbon M. Williams J. B. W. (1997). Structured Clinical Interview for DSM-IV Axis I Disorders – Patient Edition. New York: New York State Psychiatric Institute

[B26] Foxe J. J. Doniger G. M. Javitt D. C. (2001). Early visual processing deficits in schizophrenia: impaired P1 generation revealed by high-density electrical mapping. Neuroreport 12, 3815–3820 10.1097/00001756-200112040-00043 11726801

[B27] Granholm E. Fish S. C. Verney S. P. (2009). Pupillometric measures of attentional allocation to target and mask processing on the backward masking task in schizophrenia. Psychophysiology 46, 510–520 10.1111/j.1469-8986.2009.00805.x 19496224 PMC2734867

[B28] Green M. F. Lee J. Cohen M. S. Engel S. A. Korb A. S. Nuechterlein K. H. (2009). Functional neuroanatomy of visual masking deficits in schizophrenia. Arch. Gen. Psychiatry 66, 1295–1303 10.1001/archgenpsychiatry.2009.132 19996034 PMC2907419

[B29] Green M. F. Nuechterlein K. H. Breitmeyer B. Mintz J. (2006). Forward and backward visual masking in unaffected siblings of schizophrenic patients. Biol. Psychiatry 59, 446–451 10.1016/j.biopsych.2005.06.035 16139818

[B30] Green M. F. Nuechterlein K. H. Breitmeyer B. G. (2002). Development of a computerized assessment for visual masking. Int. J. Methods Psychiatr. Res. 11, 83–89 10.1002/mpr.126 12459798 PMC6878354

[B31] Green M. F. Nuechterlein K. H. Breitmeyer B. G. Mintz J. (1999). Backward masking in unmedicated schizophrenic patients in psychotic remission: possible reflection of aberrant cortical oscillation. Am. J. Psychiatry 156, 1367–1373 10484946 10.1176/ajp.156.9.1367

[B32] Green M. F. Nuechterlein K. H. Breitmeyer B. G. Tsuang J. Mintz J. (2003). Forward and backward visual masking in schizophrenia: influence of age. Psychol. Med. 33, 887–895 10.1017/S003329170200716X 12877403

[B33] Green M. F. Nuechterlein K. H. Mintz J. (1994). Backward masking in schizophrenia and mania: II. Specifying the visual channels. Arch. Gen. Psychiatry 51, 939–944 10.1001/archpsyc.1994.03950120011003 7979881

[B34] Green M. F. Wynn J. K. Breitmeyer B. Mathis K. I. Nuechterlein K. H. (2011). Visual masking by object substitution in schizophrenia. Psychol. Med. 41, 1489–1496 10.1017/S0033291710000723 21078224 PMC3266659

[B35] Jannati A. Spalek T. M. Di Lollo V. (2011). On the labile memory buffer in the attentional blink: masking the T2 representation by onset transients mediates the AB. J. Exp. Psychol. Hum. Percept. Perform. 37, 1182–1192 10.1037/a0021924 21517212

[B36] Javitt D. C. (2009). When doors of perception close: bottom-up models of disrupted cognition in schizophrenia. Annu. Rev. Clin. Psychol. 5, 249–275 10.1146/annurev.clinpsy.032408.153502 19327031 PMC4501390

[B37] Koivisto M. Revonsuo A. Lehtonen M. (2006). Independence of visual awareness from the scope of attention: an electrophysiological study. Cereb. Cortex 16, 415–424 10.1093/cercor/bhi121 15958780

[B38] Kopelowicz A. Ventura J. Liberman R. L. Mintz J. (2008). Consistency of brief psychiatric rating scale factor structure across a broad spectrum of schizophrenia patients. Psychopathology 41, 77–84 10.1159/000111551 18033976

[B39] Kotsoni E. Csibra G. Mareschal D. Johnson M. H. (2007). Electrophysiological correlates of common-onset visual masking. Neuropsychologia 45, 2285–2296 10.1016/j.neuropsychologia.2007.02.023 17452044

[B40] Lalanne L. Dufour A. Despres O. Giersch A. (2012). Attention and masking in schizophrenia. Biol. Psychiatry 71, 162–168 10.1016/j.biopsych.2011.09.018 22036035

[B41] Lamme V. A. Roelfsema P. R. (2000). The distinct modes of vision offered by feedforward and recurrent processing. Trends Neurosci. 23, 571–579 10.1016/S0166-2236(00)01657-X 11074267

[B42] Leitman D. I. Foxe J. J. Butler P. D. Saperstein A. Revheim N. Javitt D. C. (2005). Sensory contributions to impaired prosodic processing in schizophrenia. Biol. Psychiatry 58, 56–61 10.1016/j.biopsych.2005.02.034 15992523

[B43] Lukoff D. Nuechterlein K. H. Ventura J. (1986). Manual for the expanded brief psychiatric rating scale. Schizophr. Bull. 12, 578–602 10.1093/schbul/12.4.578 3810065

[B44] Maier J. Dagnelie G. Spekreijse H. van Dijk B. W. (1987). Principal component analysis for source localization VEPs in man. Vision Res. 27, 165–177 10.1016/0042-6989(87)90179-9 3576977

[B45] Overall J. E. Gorham D. R. (1962). The brief psychiatric rating scale. Psychol. Rep. 10, 799–812 10.2466/pr0.1962.10.3.799

[B46] Pinheiro A. P. Del Re E. Mezin J. Nestor P. G. Rauber A. McCarley R. W. (2012). Sensory-based and higher-order operations contribute to abnormal emotional prosody processing in schizophrenia: an electrophysiological investigation. Psychol. Med. 10, 1–16 10.1017/S003329171200133X PMC12831180 22781212

[B47] Prime D. J. Pluchino P. Eimer M. Dell’acqua R. Jolicoeur P. (2011). Object-substitution masking modulates spatial attention deployment and the encoding of information in visual short-term memory: Insights from occipito-parietal ERP components. Psychophysiology 48, 687–696 10.1111/j.1469-8986.2010.01133.x 20874751

[B48] Railo H. Koivisto M. Revonsuo A. (2011). Tracking the processes behind conscious perception: a review of event-related correlates of visual consciousness. Conscious. Cogn. 20, 972–983 10.1016/j.concog.2011.03.019 21482150

[B49] Rassovsky Y. Green M. F. Nuechterlein K. H. Breitmeyer B. G. Mintz J. (2005). Visual processing in schizophrenia: structural equation modeling of visual masking performance. Schizophr. Res. 78, 251–260 10.1016/j.schres.2005.05.011 15975768

[B50] Rassovsky Y. Horan W. P. Lee J. Sergi M. J. Green M. F. (2011). Pathways between early visual processing and functional outcome in schizophrenia. Psychol. Med. 41, 487–497 10.1017/S0033291710001054 20482936 PMC5534526

[B51] Reiss J. E. Hoffman J. E. (2006). Object substitution masking interferes with semantic processing: evidence from event-related potentials. Psychol. Sci. 17, 1015–1020 10.1111/j.1467-9280.2006.01820.x 17201780

[B52] Ro T. Breitmeyer B. Burton P. Singhal N. S. Lane D. (2003). Feedback contributions to visual awareness in human occiptial cortex. Curr. Biol. 13, 1038–1041 10.1016/S0960-9822(03)00337-3 12814549

[B53] Schechter I. Butler P. D. Zemon V. Revheim N. Saperstein A. M. Jalbrzikowski M. (2005). Impairments of early-stage transient visual evoked potentials to magno- and parvocellular-selective stimuli in schizophrenia. Clin. Neurophysiol. 116, 2204–2215 10.1016/j.clinph.2005.06.013 16055375 PMC2901806

[B54] Semlitsch H. V. Anderer P. Schuster P. Presslich O. (1986). A solution for reliable and valid reduction of ocular artifacts, applied to the P300 ERP. Psychophysiology 23, 695–703 10.1111/j.1469-8986.1986.tb00696.x 3823345

[B55] Sergi M. J. Green M. F. (2003). Social perception and early visual processing in schizophrenia. Schizophr. Res. 59, 233–241 10.1016/S0920-9964(01)00405-4 12414080

[B56] Sillito A. M. Jones H. E. Gerstein G. L. West D. C. (1994). Feature-linked synchronization of thalamic relay cell firing induced by feedback from the visual cortex. Nature 369, 479–482 10.1038/369479a0 8202137

[B57] Silverstein S. M. Keane B. P. (2011). Perceptual organization impairment in schizophrenia and associated brain mechanisms: review of research from 2005 to 2010. Schizophr. Bull. 37, 690–699 10.1093/schbul/sbr052 21700589 PMC3122298

[B58] Van Loon A. M. Scholte H. S. Van Gaal S. Van Der Hoort B. J. J. Lamme V. a. F. (2012). GABAA agonist reduced visual awareness: a masking-EEG experiment. J. Cogn. Neurosci. 24, 965–974 10.1162/jocn_a_00197 22264199

[B59] Vanni S. Warnking J. Dojat M. Delon-Martin C. Bullier J. Segebarth C. (2004). Sequence of pattern onset responses in the human visual areas: an fMRI constrained VEP source analysis. Neuroimage 21, 801–817 10.1016/j.neuroimage.2003.10.035 15006647

[B60] Ventura J. Green M. F. Shaner A. Liberman R. P. (1993). Training and quality assurance with the brief psychiatric rating scale: ‘the drift busters.’ Int. J. Methods Psychiatr. Res. 3, 221–224

[B61] Ventura J. Liberman R. P. Green M. F. Shaner A. (1998). Training and quality assurance with the structured clinical interview for DSM-IV. Psychiatry Res 79, 163–173 10.1016/S0165-1781(98)00038-9 9705054

[B62] Wilenius M. E. Revonsuo A. (2007). Timing of the earliest ERP correlate of visual awareness. Psychophysiology 44, 703–710 10.1111/j.1469-8986.2007.00546.x 17584186

[B63] Woodman G. F. Luck S. J. (2003). Dissociations among attention, perception, and awareness during object-substitution masking. Psychol. Sci. 14, 605–611 10.1046/j.0956-7976.2003.psci_1472.x 14629693

[B64] Wynn J. K. Light G. A. Breitmeyer B. Nuechterlein K. H. Green M. F. (2005). Event-related gamma activity in schizophrenia patients during a visual backward masking task. Am. J. Psychiatry 162, 2330–2336 10.1176/appi.ajp.162.12.2330 16330598 PMC9973373

